# Cannabinoid Receptor Type 1 Expression in the Developing Avian Retina: Morphological and Functional Correlation With the Dopaminergic System

**DOI:** 10.3389/fncel.2018.00058

**Published:** 2018-03-12

**Authors:** Luzia da Silva Sampaio, Regina C. C. Kubrusly, Yolanda P. Colli, Priscila P. Trindade, Victor T. Ribeiro-Resende, Marcelo Einicker-Lamas, Roberto Paes-de-Carvalho, Patricia F. Gardino, Fernando G. de Mello, Ricardo A. De Melo Reis

**Affiliations:** ^1^Laboratório de Neuroquímica, Instituto de Biofísica Carlos Chagas Filho, Universidade Federal do Rio de Janeiro, Rio de Janeiro, Brazil; ^2^Laboratório de Neurofarmacologia, Instituto Biomédico, Universidade Federal Fluminense, Niterói, Brazil; ^3^Laboratório de Biomembranas, Instituto de Biofísica Carlos Chagas Filho, Universidade Federal do Rio de Janeiro, Rio de Janeiro, Brazil; ^4^Laboratório de Neurobiologia Celular, Programa de Neurociências, Universidade Federal Fluminense, Niterói, Brazil

**Keywords:** cannabinoid, retina, development, dopamine, cAMP

## Abstract

The avian retina has been used as a model to study signaling by different neuro- and gliotransmitters. It is unclear how dopaminergic and cannabinoid systems are related in the retina. Here we studied the expression of type 1 and 2 cannabinoid receptors (CB_1_ and CB_2_), as well as monoacylglycerol lipase (MAGL), the enzyme that degrades 2-arachidonoylglycerol (2-AG), during retina development. Our data show that CB_1_ receptor is highly expressed from embryonic day 5 (E5) until post hatched day 7 (PE7), decreasing its levels throughout development. CB_1_ is densely found in the ganglion cell layer (GCL) and inner plexiform layer (IPL). CB_2_ receptor was also found from E5 until PE7 with a decrease in its contents from E9 afterwards. CB_2_ was mainly present in the lamination of the IPL at PE7. MAGL is expressed in all retinal layers, mainly in the IPL and OPL from E9 to PE7 retina. CB_1_ and CB_2_ were found both in neurons and glia cells, but MAGL was only expressed in Müller glia. Older retinas (PE7) show CB_1_ positive cells mainly in the INL and co-expression of CB_1_ and tyrosine hydroxylase (TH) are shown in a few cells when both systems are mature. CB_1_ co-localized with TH and was heavily associated to D_1_ receptor labeling in primary cell cultures. Finally, cyclic AMP (cAMP) was activated by the selective D_1_ agonist SKF38393, and inhibited when cultures were treated with WIN55, 212–2 (WIN) in a CB_1_ dependent manner. The results suggest a correlation between the endocannabinoid and dopaminergic systems (DSs) during the avian retina development. Activation of CB_1_ limits cAMP accumulation via D_1_ receptor activation and may influence embryological parameters during avian retina differentiation.

## Introduction

The retina is a unique tissue located in the posterior part of the eye involved with light transduction in visual information. The vertebrate retina, easily accessible to experimental manipulation, formed by seven major cell types, reviewed in Masland (2012), is extensively used as an experimental model to investigate cell interactions. Neurons and glia cells interact bidirectionally, and the avian retina has been used in the last 40 years, as a model to study the role of neuro-glia interactions in cell migration and development (Reis et al., 2007; de Melo Reis et al., 2008).

Dopamine is the main catecholamine found in a subtype of retinal amacrine cells located in the inner nuclear layer (INL; Reis et al., 2007). Emergence of amacrine cells begins at early stages (around embryonic day 3, E3) and is concluded at E9 (Prada et al., 1991; Calaza Kda and Gardino, 2010). Tyrosine hydroxylase (TH) expression is only found around E12, while D_1_/D_5_ type dopaminergic receptors coupled to cyclic AMP (cAMP) production are fully functional on E7 (de Mello, 1978) as compared to the end of the proliferation period (Gardino et al., 1993). Indeed, analysis of chick retina sections show TH positive dopaminergic amacrine cells located in the dorsal retina at E13/E14, and beginning to be defined at E16, with a rising arborization complexity until hatching, in a way that at E18/2 days post hatching dopaminergic cells are uniformly distributed throughout the retina (Gardino et al., 1993). In addition, specific dopaminergic amacrine cell subpopulation shows a more restrict neurogenesis period than the general amacrine cell population. However, dopaminergic system (DS) development only becomes complete in the post-embryonic phase, when TH expressing in amacrine cells in the INL are found in a highly connected wiring pattern with retinal ganglion cells, located in the ganglion cell layer (GCL), exerting a major inhibitory input. TH expression is modulated and dependent on several extrinsic and intrinsic factors that determine the dopaminergic phenotype, such as the cAMP signaling cascade activated by PACAP (Reis et al., 2007; Fleming et al., 2013). However, a clear picture of dopaminergic amacrine cell development is still not completely understood.

The endocannabinoid system (ECS) is present in almost all classes of vertebrates (Cottone et al., 2013). The modulation of cannabinoid activity is mediated by selective type-1 and type-2 cannabinoid receptors (CB_1_ and CB_2_), which are members of the G protein-coupled receptor (GPCR) family that inhibit adenylate cyclase leading to a decrease in cAMP levels. Specific endogenous endocannabinoids agonists drive these receptors. These bioactive lipids, such as anandamide (AEA) and 2-arachidonoylglycerol (2-AG), are synthesized and degraded by a limited group of enzymes presented in different cell types (Howlett, 2002). One of these enzymes, monoacylglycerol lipase (MAGL), is a serine hydrolase involved in the metabolism of 2-AG in the brain (Chanda et al., 2010).

In the CNS, CB_1_ are found mainly in presynaptic neurons, functioning as a regulatory mechanism for the release of both excitatory and inhibitory neurotransmitters (Katona and Freund, 2008).

The ECS is expressed at very early stages of the avian retinal development, as evidenced by CB_1_ expression in neurons (Leonelli et al., 2005). Recently, CB_2_ has been described in Müller glia in mammals (Bouskila et al., 2013). Straiker et al. (1999) showed for the first time that CB_1_ is expressed in the avian retina in two synaptic retinal layers, the inner plexiform and outer plexiform layers (IPL and OPL, respectively). CB_1_ expression was also reported in some amacrine and ganglion cell bodies and axons (Straiker et al., 1999). We recently showed that CB_1_ and CB_2_ receptors are found in both neurons and glial avian retinal cells in culture. These receptors regulate avian retina signaling (GABA release, calcium mobilization and cAMP levels) at critical embryonic stages during synapse formation (Kubrusly et al., 2018).

Dopaminergic neurons are modulated by ECS in different areas of the CNS. CB_1_ and endocannabinoids are abundant in the DS, acting to modulate dopamine release. It has been reported that selective and non-selective CB_1_ agonists decrease the dopamine release in the guinea-pig retina (Schlicker et al., 1996). However, the relationship between dopaminergic and ECSs during retinal development is still unclear. Therefore, we characterized the expression of CB_1_ and CB_2_ receptor, and also MAGL, during the development of avian retina as well as the possible functional correlation between the dopaminergic and ECSs in this process.

## Materials and Methods

All materials used were of analytical grade. Dulbecco’s Modified Eagle Medium: Nutrient Mixture F-12 (DMEM/F-12), fetal calf serum (FCS) and gentamycin were obtained from Gibco (USA).

### Animals

All experiments involving animals were approved by and carried out in accordance with the guidelines of the Institutional Animal Care and Use Committee of the Federal University of Rio de Janeiro (permit number IBCCF-035), and experimental procedures were carried out in accordance with the guidelines of the Brazilian Society of Neuroscience and Behavior (SBNeC). Fertilized White Leghorn eggs (*Gallus gallus*) were obtained from a local hatchery and kept in an appropriate incubator under 12 h light and 12 h dark cycles until the day of use. The eyes from chick embryos ranging from 5, 7, 9 and 14 days of incubation to 7 days post-hatching stage were used in this study. We started with embryonic day 5 (E5) since amacrine cells begin to differentiate early (E3; Calaza Kda and Gardino, 2010), CB_1_ receptors are found around E5 (Leonelli et al., 2005), and the first dopaminergic marker is described around E6 (dopamine transporter, DAT, Kubrusly et al., 2003). D_1_ receptors are found around E7 (de Mello, 1978), and amacrine neurons end their migration process (Gardino et al., 1993). A peak on retinal synaptogenesis is found around E14 (Calaza Kda and Gardino, 2010). And at P7, DS in the retina is fully differentiated (Gardino et al., 1993).

### Preparation of Tissue and Retinal Radial Sections

After decapitation, the embryos and chicks eyes were enucleated and fixed for 2 h in 4% paraformaldehyde in phosphate buffer 0.16 M, pH 7.4 (PB); after these procedures, the eyes were washed in PB three times. In 24 h, the eyes were cryoprotected in sucrose 15% and 30%, embedded in medium optimal cutting temperature (O.C.T. compound, Sakura Finetek, Torrance, CA, USA), frozen and then cut perpendicularly to the vitreal surface on a Leica CM3050 S cryostat (12 μm).

### Mixed Retinal Cells in Culture

Primary cell cultures of chick retinal cells at stage E8 (8-days embryo) were prepared as described (Paes de Carvalho and de Mello, 1982). Briefly, retinas were dissected on a Ca^2+^- and Mg^2+^-free salt balanced medium (CMF) and cleared of the pigmented epithelium. The retinas were dissociated using TrypLE^®^ (Thermo Fisher Scientific) and incubated at 37°C for 10 min. After brief centrifugation, the pellet was suspended in 1 ml of DMEM/F-12 medium supplemented with 10% FCS and mechanically dissociated with a Pasteur pipette. Cells were plated on 13 mm coverslips (Marienbad, German; 1.6 × 10^6^ cells), or 33.7 mm plates (TPP, Trasadingen, Switzerland; 20 × 10^6^ cells), previously coated with Poly-L-Lysine. The cultures were maintained in an incubator at 37°C under 5% CO_2_ atmosphere for 6 days (E8C6 cells in culture). E8C6 on 13 mm coverslips were fixed for 15 min in 4% paraformaldehyde in PBS.

### Immunofluorescence

Retina sections and primary cell cultures of retinal cells were washed three times with PBS and incubated for 30 min with 0.15% Triton X-100 and 3% FCS in PBS. This buffer was removed and the primary antibodies of interest added in an overnight incubation at 4°C. The sections and cultures were washed with PBS and incubated with fluorochrome-conjugated secondary antibody for 2 h at room temperature protecting from the light. After rinsing with PBS, the retinas and cultures were mounted using a PBS containing 40% glycerol and DAPI 0.04 μg/ml solution and were examined in a Zeiss Axio Imager 2 Fluorescence Microscope with Apotome and/or confocal microscope (LSM 510 Meta, Zeiss, Germany).

For the immunofluorescence results, specific antibodies were used according to the recommendation of each manufacturer. For primary antibodies, anti-CB1 (anti-cannabinoid receptor 1 produced in goat—SAB2500190 from Sigma-Aldrich), dilution of 1:100; anti-CB2 (CB2 antibody (M-15) produced in goat—sc-10076 from Santa Cruz Biotechnology) dilution of 1:100; anti-MAGL (anti-MAGL produced in rabbit—ab152002 from abcam) dilution of 1:100; anti-TH (anti-tyrosine hydroxylase produced in rabbit—AB152 from EMD Millipore) dilution of 1:100; anti-MAP2 (anti-MAP2 produced in mouse—ab11267 from abcam) dilution of 1:400; anti-2M6 (monoclonal anti-2M6 (kindly provided by Dr. B. Schlosshauer; Max-Planck-Institute, Tübingen, Germany, Schlosshauer et al., 1991)), dilution of 1:400; anti-D_1_ (anti-dopamine D_1R_ produced in rabbit—NBP2–16213 from Novusbio) dilution of 1:100.

For secondary antibodies, Alexa Fluor^®^ 488 donkey anti-goat (A11055 produced from Thermo Fisher Scientific); Alexa Fluor^®^ 488 donkey anti-rabbit (A21206 produced from Thermo Fisher Scientific); Alexa Fluor^®^ 594 donkey anti-rabbit (A21207 produced from Thermo Fisher Scientific); Alexa Fluor^®^ 594 donkey anti-mouse (A21203 produced from Thermo Fisher Scientific), all were used at the 1:500 dilution.

### Western Blotting

Fresh retinas from embryos and chicks, and E8C6 cells culture were washed twice with PBS, homogenized with a lysis buffer (EDTA 10 mM, HEPES-Tris 50 mM, Sucrose 1 M, trypsin inhibitor 0.15 mg/ml) and the total protein concentration was quantified (Lowry et al., 1951). Total proteins were separated by polyacrylamide gel electrophoresis (10% SDS-PAGE) and transferred to nitrocellulose membranes (Laemmli, 1970). After 1 h in blocking buffer (5% low fat dried milk in Tris-buffered saline—TBS), the immunodetection was performed by incubating the membrane with a specific primary antibody overnight at 4°C. The membranes were washed with TBS and incubated with secondary horseradish peroxidase (HRP)-conjugated antibody for 2 h at room temperature. The proteins of interest were finally detected using Luminata™ Forte (Merck Millipore) and ChemiDoc MP system (Bio-Rad).

For the western blotting results, we used specific antibodies, according to the recommendation of each manufacturer. For primary antibodies, anti-CB_1_ (anti-cannabinoid receptor CB_1_ (1–77) produced in rabbit–209550 from Calbiochem) was used at a dilution of 1:1000; anti-CB_2_ (anti-CNR2 antibody produced in mouse—WH0001269M1 from Sigma-Aldrich) was used at a dilution of 1:1000; anti-TH (anti-tyrosine hydroxylase produced in rabbit—AB152 from EMD Millipore) was used at a dilution of 1:500; anti-D_1_ (anti-dopamine D_1R_ produced in rabbit—NBP2–16213 from Novusbio) was used at a dilution of 1:500; anti-DARP32 (anti-DARPP-32 antibody [EP720Y] produced in rabbit—ab40801 from abcam) was used at a dilution of 1:500; anti-Nurr-1 (anti-Nurr-1 antibody produced in rabbit—ab93332 from abcam) was used at a dilution of 1:500; anti-ERK1/2 (anti p44/42 MAPK (Erk1/2) antibody produced in mouse–4696 from cell signaling) was used at a dilution of 1:1000; anti-β actin (anti-β-actin clone AC-15 produced in mouse—A5441 from Sigma-Aldrich) was used at a dilution of 1:5000. For secondary antibodies, HRP-conjugated goat anti-mouse (A5278 from Sigma-Aldrich) and HRP-conjugated goat anti-rabbit (A0545 from Sigma-Aldrich) were used at a dilution of 1:5000 (for the β actin protocol, we used the dilution 1:25,000). For statistical analysis, densitometry for each protein of interest was performed using Scion Image software followed by ANOVA.

### cAMP Accumulation

Accumulation of cAMP was assayed according to the competitive binding assay as described previously (Kubrusly et al., 2005). Mixed retinal cells were washed with PBS and pre-treated with 5 × 10^−4^ M IBMX, an inhibitor of phosphodiesterases, for 15 min in 1 ml of DMEM/F-12 plus 20 mM HEPES pH 7.4 at 37°C. To measure adenylyl cyclase activity, we used 5 × 10^−5^ M SKF38393, a D_1R_ agonist, for 30 min at 37°C, or 1 × 10^−7^ M forskolin, a general activator for this enzyme. The treatments performed were 10^−6^ M AM251, a CB_1_ antagonist, for 15 min; 10^−6^ M WIN55, 212–2, a non-selective cannabinoid receptor agonist, for 30 min. The reaction was stopped by addition of 100 μl trichloroacetic acid 100% (5% final) acid followed by storage at −20°C for 24 h. After centrifugation (1000 *g* for 10 min), the supernatant was passed through an ion-exchange resin column (Dowex 50) to remove the trichloroacetic acid and other nucleotides. The samples containing cAMP were then incubated in the presence of the regulatory subunit of PKA and a fixed trace amount of [^3^H]cAMP in 50 mM acetate buffer, pH 4.0, at 4°C for 90 min. The reaction was interrupted by the addition of 200 mM phosphate buffer at pH 6.0. The samples were filtered through Millipore filters and the radioactivity determined in a Tri Carb 2810 TR Perkin Elmer liquid scintillation analyzer.

### Statistical Analysis

All the results are shown as means ± SEM. Graphpad Prism5^®^ software was used to analyze the results by one way ANOVA, followed by Tukey post-test. For all the results, *p* < 0.05 was taken as the significance level, and the number of experiments is described in each figure legend.

## Results

**Figure 1:**
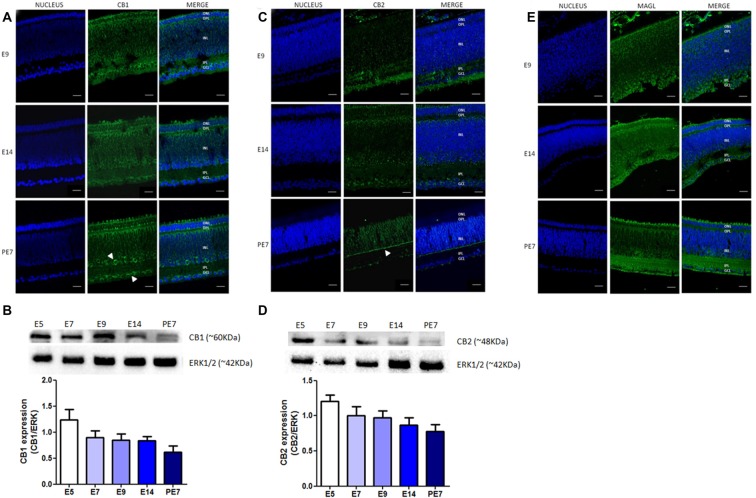
Expression of selective endocannabinoid markers during avian retina development. Expression of CB_1_, CB_2_ and monoacylglycerol lipase (MAGL) using immunofluorescence and western blotting. In **(A)**, expression of CB_1_ (green) at embryonic stages E9 and E14 and post-hatched 7 days (PE7) in chick retina. At PE7, CB_1_ is mainly found in cells of the inner nuclear layer (INL) and ganglion cell layer (GCL; white arrows). **(B)** Western blot highlights CB_1_ expression from embryonic day 5 (E5) to PE7. Expression of ERK 1/2 was used as loading control. In **(C)**, CB_2_ (green) expression from E9 to PE7 primarily in the inner plexiform layer (IPL) lamina (white arrow). CB_2_ expression from E5, as shown in **(D)**. In **(E)**, MAGL expression (green) from E9 to PE7 is shown, its distribution is evident across all layers of the retina. Scale bar of 20 μm, all images were obtained with 40× magnification in a Zeiss fluorescence microscope. *N* = 6 for each analysis.

Our results confirm that CB_1_ receptor is highly expressed in the chick retina. Indeed, we analyzed its expression during ontogenesis from embryonic day 9 (E9) for immunofluorescence and E5 for western blotting, until post-hatched day 7 (PE7; Figures 1A,B). Western blotting data show a decrease, but not statistically different, in the CB_1_ expression during avian retinal development (Figure 1B). CB_1_ is located in almost all retinal layers from E14 to PE7, and is more densely present in the GCL and IPL (Figure 1A).

We also evaluated the expression of other ECS proteins, such as CB_2_ receptor and MAGL, the enzyme that degrades the endocannabinoid agonist 2-AG. Our data clearly show the expression of CB_2_ receptor throughout development, between E5 and PE7 retinas (Figure 1D). We also observed a decrease in CB_2_ protein content from E5 afterwards and that these receptors are mainly present in the lamination of the IPL at PE7 retinas (Figure 1C). MAGL is expressed in all retinal layers, but more clearly in the IPL and OPL from E9 to PE7 retina (Figure 1E).

**Figure 2:**
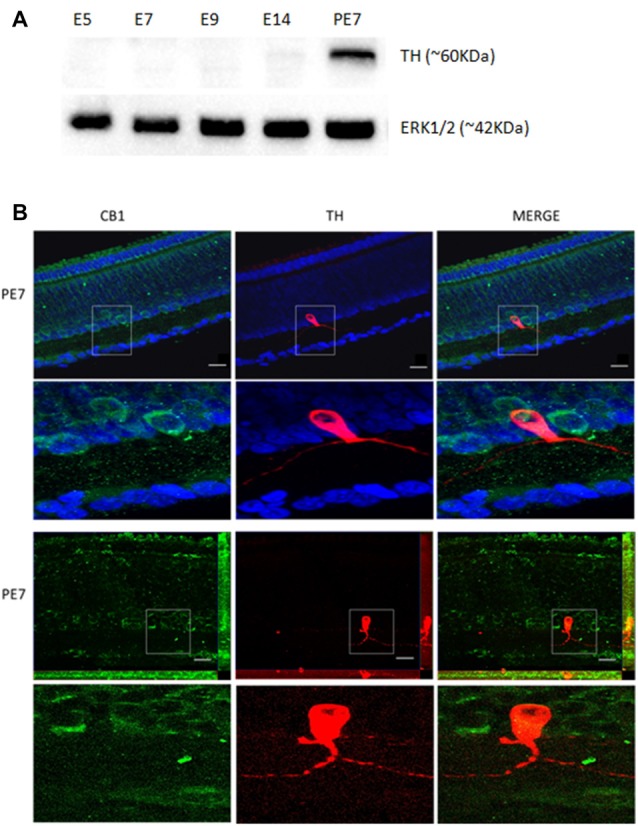
Co-localization of CB_1_ receptor and tyrosine hydroxylase (TH) enzyme in the avian retina. We evaluated whether dopaminergic neurons, TH positive cells (red) express CB_1_ receptor (green) by immunofluorescence. In **(A)**, western blotting for TH at different stages, confirming that TH is highly expressed at PE7. ERK1/2 is shown as the loading control. In **(B)**, CB_1_ and TH co-expression in PE7 avian retina. As shown, a discrete TH co-expression with CB_1_ that is mainly present in neighboring cells in the INL. ApoTome images in upper row and confocal images in lower row. Scale bar of 20 μm, all images were obtained with 40× magnification. *N* = 5 for each analysis.

To test our hypothesis of a link between the ECS and the DS, we asked whether the CB_1_ receptor is co-expressed with TH in retina dopaminergic cells. We performed western blotting for TH at different stages, confirming that TH is highly expressed at PE7 (Figure 2A). TH amacrine neurons express CB_1_ receptor in the INL and GCL cells around PE7 (Figure 2B). Indeed, CB_1_ positive cells mainly in the INL are found co-expressed with a few TH positive cells (Figure 2B).

**Figure 3:**
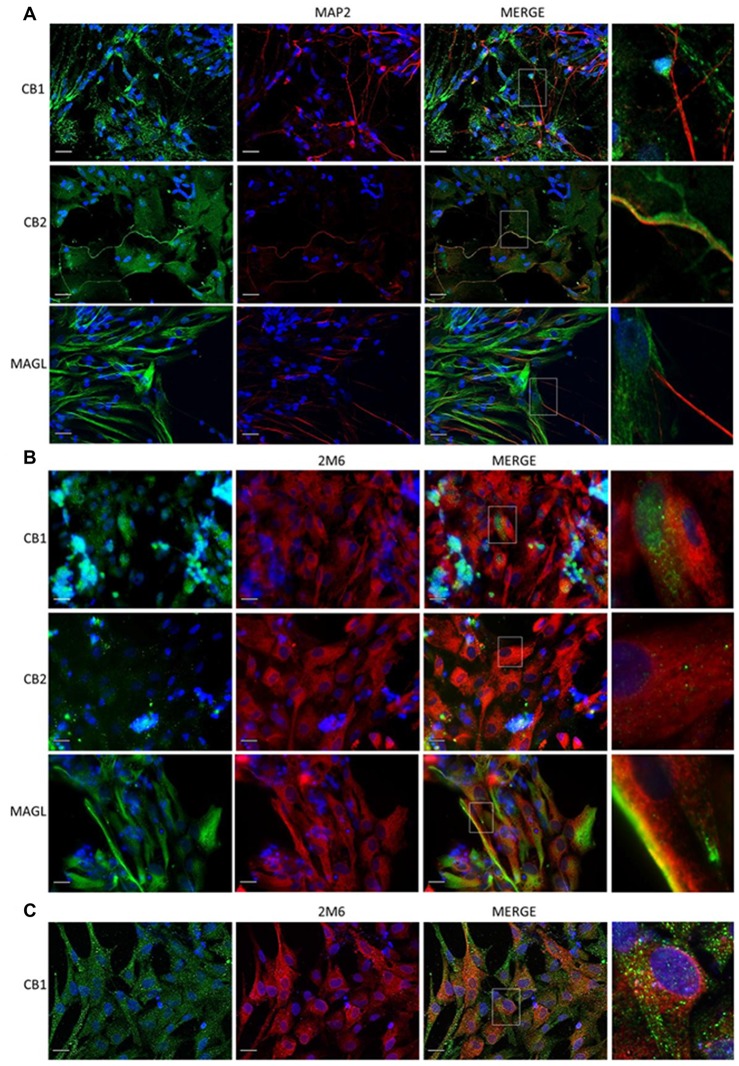
Expression of cannabinoid markers in primary retinal cell in cultures. Expression of CB_1_, CB_2_ and MAGL in mixed cultures of avian retina cells, displaying neurons and glia prepared from 8-day old embryos (E8), as described. In **(A)**, CB_1_, CB_2_, MAGL (all in green) and MAP2 (red) as a specific neuronal marker. In **(B)**, expression of CB_1_, CB_2_, MAGL (green) and 2M6 (red) as a selective glial marker for Müller cells. In **(C)**, purified culture of Müller cells show expression of CB_1_ (green). Scale bar of 20 μm, all images were obtained with 40× magnification in a fluorescence microscope with ApoTome software. *N* = 5.

Since dopaminergic neurons express CB_1_ receptor, we decided to investigate whether this receptor modulate dopaminergic functions in the avian retina. For this purpose, we used primary cell cultures of mixed neurons and Müller glia (Figure 3), as these cells express both CB_1_ and CB_2_ receptors (Kubrusly et al., 2018) and also MAGL. CB_1_ and CB_2_ are found both in neurons and glia cells, but MAGL is only expressed in Müller glia. CB_1_ is highly expressed in neuron bodies (Figure 3), and in Müller cells purified culture (Figure 3C).

**Figure 4:**
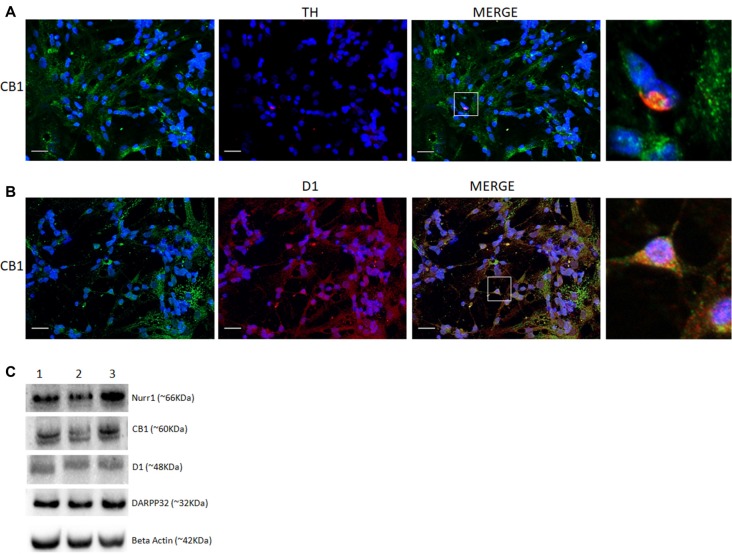
Co-expression of selective cannabinoid and dopaminergic markers in cultured retinal cells. Expression of CB_1_ receptor in E8C6 cultures showing the main components of the dopaminergic system (DS). In **(A)**, co-localization of CB_1_ (green) in TH positive neurons (red). In **(B)**, co-localization of CB_1_ (green) in cells expressing the D_1_ receptor (red). In **(C)**, western blotting analyses confirm the expression dopaminergic markers (Nurr1 and Darpp32) and D_1_ receptor, in addition to CB_1_ expression, β Actin is shown as the loading control (lanes represent triplicate assay as shown for 1, 2, 3). Scale bar of 20 μm, all images were obtained with 40X magnification in a fluorescence microscope with ApoTome software. *N* = 3 for each analysis.

CB_1_ was also found to be co-localized with TH and heavily associated with D_1_ receptor labeling in primary cell cultures (Figures 4A,B). To explore the presence of selective dopaminergic markers in mixed retinal cell cultures, we performed western blot analysis of different proteins associated with the DS. Data revealed the clear expression of the nuclear receptor related 1 protein (Nurr1), D_1_ receptor and dopamine- and cAMP-regulated phosphoprotein 32 (Darpp32; Figure 4C) in addition to CB_1_.

**Figure 5:**
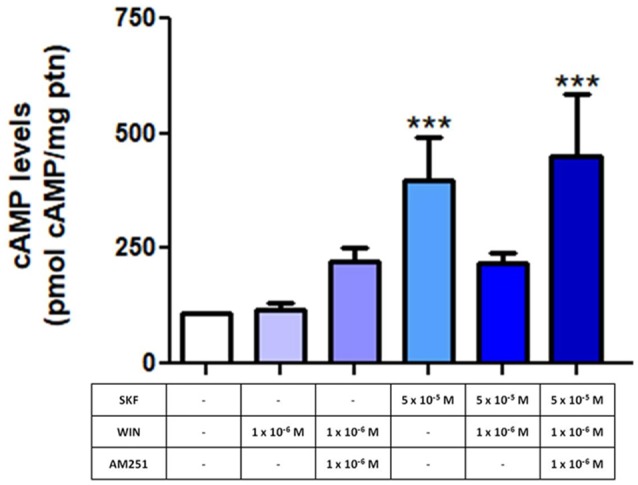
Endocannabinoid system (ECS) modulates the function of dopamine-induced increase in cyclic AMP (cAMP) levels. E8C6 retinal mixed cell culture was subjected to cAMP quantification analysis, as described. When the selective D_1_ receptor agonist, SKF38393 (SKF) at 5 × 10^−5^ M was used, cAMP levels increased significantly. When the culture was treated with WIN after SKF stimulation, the SKF-induced increase in cAMP levels was lost. However, in cultures pretreated with AM251, a selective CB_1_ antagonist, cAMP levels were increased (AM251 + SKF + WIN), similar to SKF-only application. AM251 was added 15 min before SKF and WIN, which were added for 30 min, all at 37°C. ***< 0.001. *N* = 6 for each analysis.

Next, E8C6 cultures were exposed to SKF38393 (SKF), a selective D_1_ agonist, which was able to increase the cAMP levels when compared to controls. Our results show that the cAMP accumulation induced by SKF is lost when cultures were pretreated with WIN55, 212–2 (WIN), a non-selective CB agonist (Figure 5). When cultures were pre-treated with AM251, a CB_1_ selective antagonist, and then co-exposed to SKF+WIN, the SKF response was restored (Figure 5), indicating that the repressive effect of WIN on SKF promoted cAMP accumulation was due, mainly, to activation of CB_1_ receptors.

## Discussion

Here we show the expression of CB_1_ receptors in the avian retina throughout development, being detected since an early embryonic stage (E5) up to a more mature period (PE7). Moreover, we show a decrease in CB_1_ protein content during development, but no significance was reached upon ANOVA. CB_1_ is mainly located in ganglion as well as amacrine cells, present in the GCL and in the INL, respectively. Our work confirms previous studies that have shown the presence of CBs and endocannabinoids metabolic enzymes in the retina of several species such as goldfish, rat, mouse, chick and monkey (Schwitzer et al., 2016). Some of these previous data have shown CB_1_ expression in ganglion, horizontal and amacrine cells, cone pedicles and rod spherules of photoreceptors (Zabouri et al., 2011a). Leonelli et al. (2005) showed for the first time that, in avian retina, CB_1_ emerges in E4, in ganglion cells, while at E18 CB_1_ is expressed in neurons located in the GCL and INL. We recently showed that CB_1_ and CB_2_ receptors are found in both retinal neurons and Müller glia of chick embryos. We found that WIN decreases cAMP production in retinal cells in basal conditions, decreases the number of glial cells that increased Ca^2+^ evoked by ATP, and inhibited [^3^H]-GABA release induced by KCl or L-Aspartate (Kubrusly et al., 2018). Now we evaluated the expression of CB_1_, CB_2_ receptor and MAGL enzyme in the developing retina. CB_2_ has been reported in rat, mouse and monkey but not in avian retina. CB_2_ has been shown to be expressed in photoreceptors, horizontal, amacrine and ganglion cells and also in fibers in the INL in adult rat retina (López et al., 2011). Interestingly, CB_2_ expression appears in Müller cells in the monkey retina (Bouskila et al., 2013). However, there is no information regarding CB_2_ expression during development of the avian retina. Our data show that CB_2_ is expressed beginning at E5 and is clearly observed in the lamination of the IPL between E14 and PE7 (Figure 1C). In early retinogenesis, up to E5, retinal cells are essentially in the neuroblastic layer made of multipotent progenitors that generate early-born cells (RGCs, horizontal, some types of amacrines and cones). Around E14, late-born progenitors give rise to bipolar, rods and Müller glia, so the precise number and proportion of cells into different layers form a fully mature retina (Martins and Pearson, 2008). Since CB_1_ (Xapelli et al., 2013) and CB_2_ (Bravo-Ferrer et al., 2017) receptors are correlated with proliferation and/or neurogenesis in the central nervous system, their presence early in the embryonic avian retina could modulate the generation of retinal cells. Moreover, activation of these receptors at early stages suggests that the retinal circuitry might be altered (morphologically) or in terms of function (CBs inhibit the release of GABA and aspartate (Kubrusly et al., 2018), and decrease cAMP levels, as shown in Figure 5. This could possibly reflect in changes during synapse remodeling during retinal development.

The main endocannabinoids are AEA and 2-AG, described in the retina of several vertebrates along with the main enzymes that degrade them (Schwitzer et al., 2016). MAGL, one of these enzymes, is present in rat retinal layers mainly in amacrine and Müller cells (Cécyre et al., 2014). We found that, in the avian retina, MAGL is expressed beginning at E9 and up to post-hatched PE7, where it is detected in all the five retinal layers, mostly in IPL and GCL (Figure 1E). Our data provide that MAGL is located primarily in the glial compartment as the enzyme is co-localized with 2M6 (Figure 3B, lower panel), at least *in vitro*. As glial cells are the last to be generated in the retina (Martins and Pearson, 2008), perhaps this explains the appearance of labeling at E9. We still cannot affirm how the developmental profile of CB_1_, CB_2_, MAGL and endocannabinoids match the interactions between different retinal neurons and Müller glia. However, data on Figure 5 suggest an important interplay between receptors that decrease the levels of cAMP (CB_1_ and CB_2_) and those that increase cAMP levels (dopamine, adenosine or PACAP) during retina differentiation.

In addition, CB receptor activation decreases the number of glial cells that increased Ca^2+^ evoked by ATP, and inhibited [^3^H]-GABA release induced by KCl or L-Aspartate (Kubrusly et al., 2018). The first part of the retina that differentiates is the central part and RGCs are the first to emerge. Synaptogenesis peak in the avian retina around E14, and it is known to have two main axis: glutamatergic in the vertical axis (photoreceptors, bipolar and the RGCs) and GABAergic in the horizontal axis (horizontal and amacrine; Barnstable, 1993). Moreover, Müller glia is an active compartment that shapes the circuitry (de Melo Reis et al., 2008). The ECS seems to be a powerful regulator of the efficacy of retinal circuitry.

The retina presents the main neurotransmitters found in the brain such as glutamate (Connaughton, 1995), GABA (Bringmann et al., 2009) and dopamine (Reis et al., 2007). A direct action of the ECS on retinal transmitter release and in general retinal physiology has been described in different vertebrate retinas mainly in retinal bipolar cells (Straiker et al., 1999). Interestingly, dopamine and noradrenaline release is inhibited by CB_1_ activation in perfused guinea-pig retina (Schwitzer et al., 2016). In the developing vertebrate retina, the DS is one of the first phenotypes to appear mainly in amacrine and interplexiform cells (Reis et al., 2007). Here, we first evaluated the emergence of CB_1_ and TH co-expression in PE7 avian retina. We observed that CB_1_ is co-localized with dopaminergic neurons (Figure 2) present in the IPL. CB_1_ is also localized in neighboring cells in IPL and also GCL, suggesting a more complex role in retina signaling. It is likely that CB_1_ positive cells are responsive for the dopamine released by TH amacrine cells and vice versa. Bosier et al. (2007) showed an important relationship between the ECS and DS using a murine neurospheres model, where CB_1_ agonists promoted a reduction in TH expression.

It is known since the 70s that dopamine increases cAMP levels in the chick retina and in mixed cultures of embryonic chick retina cells (de Mello, 1978). Since it is described that CB_1_ expression occurs in retinal cells in response to dopaminergic stimuli (Fan and Yazulla, 2005), we investigated the correlation between ECS and the DS. Primary cell cultures of mixed neurons and Müller glia express CB_1_ and CB_2_ receptors as well as MAGL enzyme. CB_1_ is predominantly in cell bodies while CB_2_ is found mainly in neuronal processes. On the other hand, MAGL is mainly located in Müller cells.

In order to evaluate a functional correlation between CB_1_ and D_1_ receptors, a mixed neuron-glia culture was used at E8C6, a stage where most of dopaminergic proteins are expressed. The D_1_ receptor agonist SKF38393 promotes a significant increase in cAMP accumulation in retina cells (Castro et al., 1999). In order to test the effect of the ECS on this D_1_-mediated effect, we added AM251, a CB_1_ receptor selective antagonist, and WIN 55, 212–2 (WIN), a non-selective cannabinoid CB_1_/CB_2_ agonist. Our results show that WIN decreases cAMP accumulation induced by SKF, and this response is blocked when AM251 was used as a pretreatment. Additionally, WIN did not change cAMP accumulation promoted by forskolin (not shown), a general adenylyl cyclase activator, suggesting that WIN is selectively acting on dopaminergic receptor level. Therefore, we provide evidence here that CB_1_ receptor can modulate the response of D_1_ receptor activation through cAMP accumulation. We conclude that the endocannabinoid and the DS interact in retinal cells.

Emergence of the ECS (CB_1_/CB_2_ receptors), endocannabinoids and enzymes during embryonic retinal development might influence dopaminergic communication that seems to use two separated systems during development. One operates in early stages of retina formation, and the other, later in development. No TH is detected in the tissue between E5 and E10/12. However, E5 retinas display several cells (neuroblasts) clearly labeled for L-Dopa decarboxylase (DDC; da Silva et al., 2009). At this stage, or even before, the presumptive neuroretina is already associated with the pigmented epithelium, characterized by the presence of melanin. Melanin, as dopamine, use as precursor L-Dopa that in the epithelium comes from tyrosinase activity. Our group has shown that conditioned media from the pigmented epithelium of embryonic eyes fed to embryonic retina tissue prior to TH expression, is capable, of synthesizing dopamine (Kubrusly et al., 2003). This activates cAMP formation prior to TH expression. Therefore, based on the findings that D_1_ (increasing cAMP) or D_2_ receptors activation (decreasing cAMP levels) in embryonic avian retina can modify the dynamics of neurites outgrowth, interference with these systems via CBs might influence dopaminergic circuitry. D_1_ and CB_1_ have been shown to exhibit antagonistic effects in goldfish bipolar retinal cells, as D_1_ activation increases cAMP level, while CB_1_ (WIN) reduces the concentration of cAMP (Fan and Yazulla, 2005). Our data strengthen this point using avian retinal cells to assess the interaction between the endocannabinoid and DSs.

A recent article has revealed in a pioneer way the presence of both cannabinoid receptors (CB_1R_ and CB_2R_, the synthesizing and degrading enzymes in mice, shrews and monkey retinae (Bouskila et al., 2016). These data, added to ours in chicks (and many others) suggest a strong conservation of the ECS in the retinal tissue in vertebrates. This opens a possibility to create strategies to ameliorate vision problems with cannabinoid modulators. A recent article has shown that activation of CB_2_ receptors with a selective agonist (JWH-133) increases inflammation in human retinal pigment epithelium, decreasing cellular viability through release of pro-inflammatory cytokines and might be a central element in avoiding vision problems (Hytti et al., 2017). Both neuronal and glial CB_1_ and CB_2_ receptors might have an important role in retinal development through modulation of intracellular pathways (Kubrusly et al., 2018). Indeed, 2-AG is found in early retinal development together with DAGLα, 2-AG degrading enzyme), while MAGL, develops later (Cécyre et al., 2014). FAAH, (AEA, degrading enzyme) emerges transiently at postnatal day 1 in ganglion and cholinergic amacrine cells (Zabouri et al., 2011b).

Moreover, we show that endocannabinoids may act on the developing CNS (here exemplified by the retina), possibly influencing developmental characteristics of CNS systems such as plasticity during synapses formation (Fleming et al., 2013). We also call attention to the possibility that cannabinoid consumption might have a dramatic effect in the developing CNS.

## Author Contributions

LSS and RCCK: conception and design, provision of study material, collection and assembly of data, data analysis and interpretation, manuscript writing and final approval of manuscript. YPC, PPT, VTR-R, ME-L and PFG: collection and assembly of data, data analysis and interpretation. RP-C, FGM and RAMR: conception and design, provision of study material, assembly of data, data analysis and interpretation, manuscript writing, final approval of manuscript, financial support and administrative support.

## Conflict of Interest Statement

The authors declare that the research was conducted in the absence of any commercial or financial relationships that could be construed as a potential conflict of interest.
